# CMV meningitis associated with dimethyl fumarate therapy-induced lymphopenia in a multiple sclerosis patient

**DOI:** 10.1007/s00415-021-10661-z

**Published:** 2021-06-27

**Authors:** Ann-Kathrin Kogel, Ralf Gold, Ruth Schneider

**Affiliations:** Department of Neurology, St. Josef-Hospital, Ruhr-University Bochum, Gudrunstr. 56, 44791 Bochum, Germany

Dear Sirs,

A 55-year-old Caucasian man with relapsing-remitting multiple sclerosis (RRMS) presented to hospital with headache, neck pain, nausea and chills. Symptoms started 3 hours prior to his presentation. Clinical examination showed meningism. MS was diagnosed in 2018 and was currently treated with dimethyl fumarate (DMF) (480 mg/day) for the last 9 months (4/20–1/21). Recommended white blood cell controls had not been realized in the last 6 months by the outpatient physicians. Previous MS therapy was 300 mg ocrelizumab in March 2018, after one infusion this therapy was stopped on patients’ request. His medical history included type 2 diabetes mellitus (HbA1c 6.4%; normal value: 4.8–6.0), hypertension and coronary artery disease.

Upon admission, blood count showed a normal white cell count (9480/µl) but marked lymphopenia (240/µl). C-reactive protein was 18.40 mg/l (normal value < 5 mg/l), procalcitonin was 4.68 ng/ml (normal value 0.5 ng/ml). U-Status showed a urinary tract infection (UTI), blood cultures detected E.coli in 4/4 cultures. Lumbar puncture revealed clear cerebrospinal fluid (CSF), investigations presented 3 white blood cells (WBC)/µl with mildly raised lactate (29 mg/dl; normal value 10-22 mg/dl), normal protein (25 mg/dl; normal value 15-45 mg/dl) and glucose levels (CSF 115 mg/dl, serum 246 mg/dl). Multiplex-polymerase chain reaction (PCR) of the CSF was positive for cytomegalovirus (CMV), further CMV-PCR amplification detected 46000copies/ml. CMV serology was positive for anti-CMV IgG (68.00U/ml; normal value < 0,5U/ml), while anti-CMV IgM and CMV-PCR in serum were negative. Brain MRI did not show any signs of encephalitis or vasculitis in time-of-flight (TOF)—angiography. Serological testing for hepatitis B and C and human immunodeficiency virus (HIV) was negative.

Given the clinical picture the diagnosis was CMV meningitis and urosepsis. DMF therapy was immediately stopped. The patient was started on intravenous ganciclovir 600 mg two times a day for 16 days. Lumbar puncture was performed on day 4, 11 and 15 and showed a significant decrease of CMV-DNA count (day 4 18,600 copies/ml, day 11 2000 copies/ml, day 15 no CMV-DNA detectable). Urosepsis was simultaneously treated with ceftriaxone and the patient gradually improved.

Worldwide CMV seroprevalence is estimated around 40–100% in the general population. In immune-competent hosts primary infection is usually asymptomatic or unspecific, e.g., fever or respiratory symptoms [1]. Persons with immune deficiency may suffer a severe disease course with pneumonia, meningitis or encephalitis [1, 2]. In the context of MS therapies, CMV infections were described during natalizumab [3] and alemtuzumab [4] treatment.

DMF is an oral disease-modifying therapy with potential immunomodulatory effects approved for patients with RRMS. In the phase 2b/3/long-term studies absolute lymphocyte count (ALC) decreased by 30% during the first year of DMF treatment followed by stabilization [5]. Regarding immune cell subsets, the relative frequencies of circulating memory T- and B-cell populations declined and naive cells increased. CD4 and CD8 T-lymphocyte counts closely correlated with ALC [6]. Increased incidence of serious infections was not observed in the long-term extension studies [5, 7].

To our knowledge, CMV meningitis under DMF treatment has not been reported yet. In view of positive CMV-specific PCR in CSF and IgG but not IgM antibody response, a reinfection or reactivation has to be assumed. The setting of immunosuppression and E.coli associated UTI with CMV reactivation was further described in the context of kidney transplants [8]. Other potential risk factors than DMF could be previous ocrelizumab therapy and diabetes mellitus. Since ocrelizumab was stopped almost three years prior and HbA1c was 6,4%, we considered these factors less likely to be causative, but cannot completely exclude it. Reduced immune response with moderate WBC-elevation and lymphopenia even in the context of systemic infection may explain absence of pleocytosis despite presence of CMV-meningitis. In summary, we aim to point out that lymphopenia is an important side effect of DMF treatment which needs to be watched closely especially in the 1st year after starting treatment.

## Data Availability

Not applicable.

## Abbreviations

ALC: Absolute lymphocyte counts
CMV: Cytomegalovirus
CSF: Cerebrospinal fluid
DMF: Dimethyl fumarate
HIV: Human immunodeficiency virus
PCR: Polymerase chain reaction
RRMS: Relapsing–remitting multiple sclerosis
TOF: Time-of-flight
UTI: Urinary tract infection
WBC: White blood cells
